# Mediterranean Diet and Type 2 Diabetes Mellitus: A Perpetual Inspiration for the Scientific World. A Review

**DOI:** 10.3390/nu13041307

**Published:** 2021-04-15

**Authors:** Tatjana Milenkovic, Nadica Bozhinovska, Djuro Macut, Jelica Bjekic-Macut, Dario Rahelic, Zelija Velija Asimi, Azra Burekovic

**Affiliations:** 1 Diabetes and Metabolic Diseases, University Clinic of Endocrinology, 1000 Skopje, North Macedonia; 2 Medical Faculty, University “St. Cyril and Methodius”, 1000 Skopje, North Macedonia; 3 Department of Endocrinology, Private Clinical Hospital “Acibadem Sistina”, 1000 Skopje, North Macedonia; nadica.bozhinovska@acibademsistina.mk; 4 Clinic of Endocrinology, Diabetes and Metabolic Diseases, Faculty of Medicine, University of Belgrade, 11000 Belgrade, Serbia; djmacut@gmail.com; 5 Department of Endocrinology, CHC Bezanijska Kosa, Faculty of Medicine, University of Belgrade, 11000 Belgrade, Serbia; jbjekic@yahoo.com; 6 “Vuk Vrhovac” University Clinic for Diabetes, Endocrinology and Metabolic Diseases, “Merkur” Univeristy Hospital, 10000 Zagreb, Croatia; dario.rahelic@gmail.com; 7 School of Medicine, University of Zagreb, 10000 Zagreb, Croatia; 8 School of Medicine, Josip Juraj Strossmayer University of Osijek, 31000 Osijek, Croatia; 9 Sarajevo Medical School, SSST University, 71210 Sarajevo, Bosnia and Herzegovina; zelijav@gmail.com; 10 Outpatient Clinic “Altamedica-Beta”, Zmaja od Bosne 7, 71000 Sarajevo, Bosnia and Herzegovina; 11 Faculty of Medicine, Sarajevo University, 71000 Sarajevo, Bosnia and Herzegovina; azraburekovic@hotmail.com; 12 Department of Endocrinology and Diabetes, Clinical Center of Sarajevo University, 71000 Sarajevo, Bosnia and Herzegovina

**Keywords:** type 2 diabetes mellitus, Mediterranean diet, obesity, insulin resistance, prediabetes

## Abstract

For the past 80 years, the effect of the Mediterranean diet on overall health has been a constant topic of interest among medical and scientific researchers. Parallel with the persistent global rise of cases of type 2 diabetes, many studies conducted in the past 20 years have shown the benefits of the Mediterranean lifestyle for people with, or at risk of developing, type 2 diabetes mellitus. However, despite the large body of evidence, concerns exist amongst scientists regarding the reliability of the data related to this topic. This review offers a glimpse of the onset of the Mediterranean diet and follows its significant impact on the prevention and treatment of type 2 diabetes. There is a constant rise in type 2 diabetes cases on the Balkan Peninsula and North Macedonia in particular. Having in mind that North Macedonia, as well as most of the countries on the Balkans have low to middle income, there is a need for a certain affordable dietary pattern to ameliorate the rise in diabetes incidence, as well as improve the glycemic control. We did a review based on the available literature regarding Mediterranean diet and people with or at risk of developing type 2 diabetes mellitus, its effects on glycemic control, lipid profile and metabolic outcome.

## 1. Introduction

It is now well known that people with diabetes are prone to chronic micro- and macrovascular complications that affect their quality of life. In addition, people with diabetes have shortened life expectancies [1] and a concomitant and doubled risk of developing cardiovascular disease and cognitive impairment [2,3,4]. Previous studies have also shown that individuals with diabetes have notably increased probability of developing certain types of cancer [1,5,6].

### 1.1. Type 2 Diabetes Treatment—New Treatment Opportunities and Prevention Management

The treatment of type 2 diabetes has improved in quality and quantity in the past two decades. Pharmacological approaches have notably been enhanced; therefore, physicians have different choices of applicable medications. As maintained by the American Diabetes Association (ADA) 2020 diabetes guidelines, the treatment for type 2 diabetes includes specific criteria concerning the expected efficacy of the therapy, the possible side effects, cost, and effects on body weight, in addition to certain individual patient factors such as cardiovascular risk, microvascular complications, and patient preferences [7]. In recent years, there has been a sharp increase in glucagon like peptide 1 (GLP-1) receptor agonists, sodium-glucose co-transporter-2 (SGLT2) inhibitors, thiazolidinediones, and dipeptidyl peptidase 4 (DPP-4) inhibitors. Nonetheless, despite this significant pharmacological improvement, the Standards of Medical Care in Diabetes 2020 by the ADA includes a separate section that focuses on lifestyle modifications that improve diabetes and, consequently, overall health [7,8,9]. The Finnish Diabetes Prevention Study (DPS) was one of the first controlled, randomized studies to show that type 2 diabetes is preventable with lifestyle intervention [7]. A 58% reduction in diabetes has been found compared to the control group [10]. In addition, these results were supported by the Diabetes Prevention Program (DPP), which showed a similar risk reduction, in addition to superior outcomes compared to metformin treatment [11]. These studies showed that type 2 diabetes is largely preventable and treatable via behavioral alterations and modifications. At present, lifestyle modification remains the healthiest and theoretically easiest recommendation, however the adherence to a healthy lifestyle and proper nutritional choices are difficult to achieve for people with type 2 diabetes.

### 1.2. Lifestyle Modifications in the Treatment of Diabetes

The International Diabetes Federation (IDF) has classified individuals who are overweight or obese, with an unhealthy diet, with a sedentary way of life, and/or with a family history of diabetes as a risk category with a strong predisposition towards type 2 diabetes mellitus [12]. An unhealthy lifestyle clearly contributes to diabetes development. Thus, the next logical step in the management of an incurable disease is to take preventative action, in addition to providing treatment in the early stages [13]. Healthy and efficacious lifestyle modifications, including habit management, regular physical activity, and mental stability, are recommended to achieve treatment objectives for people with diabetes [8,9].

## 2. The Revolution of the Primary Prevention of Cardiovascular Disease with a Mediterranean Diet (PREDIMED)

### 2.1. The Uprising of Healthy Diets

Many patients newly diagnosed with diabetes face difficulties when choosing their next meal. Some of them ask for a generic recommendation and become anxious when they learn that there is no generic solution that suits all patients. Although a single food solution does not exist for each patient, there is a general consensus among physicians that an individualized eating plan based on a certain type of food can result in significant reduction of the glycated hemoglobin A1c (HbA1c) [14,15]. Over the past decennary, an increasing number of studies have focused on certain diet benefits regarding diabetes [16,17,18]. Nonetheless, more research into the lifestyle-related benefits for diabetes prevention is needed [16].

### 2.2. The Mediterranean Diet as an Important Key Factor in Metabolic Outcomes

The Primary Prevention of Cardiovascular Disease with a Mediterranean Diet (PREDIMED) study started a revolution by finally providing important evidence of the healthfulness of a certain dietary pattern [19], in addition to showing that certain types of food can positively affect the metabolism. However, the study was retracted due to certain technicalities and protocols, which provided an opportunity for those who doubted the study’s findings to express their concerns. However, since it was republished, the PREDIMED study has made an extremely important contribution to modern medicine [19]. This study showed that among those individuals assigned to a Mediterranean diet supplemented with extra-virgin olive oil or nuts the rate of major cardiovascular events was lower, compared to the group with the more popular diet of reduced fat. In addition, PREDIMED showed that a Mediterranean diet with nuts helped 13.7% of people with metabolic syndrome reverse their condition [19]. In addition to these data, for the first time, the PREDIMED study firmly indicated that the Mediterranean diet reduced the risk of developing type 2 diabetes by 52% in patients who had no diabetes at the beginning of the study [20,21,22]. Due to the magnitude of the effect of PREDIMED in medical circles, the quest for the perfect dietary pattern for diabetes began following the publication of this study.

## 3. Mediterranean Diet Is a Lifestyle

**Figure 1:**
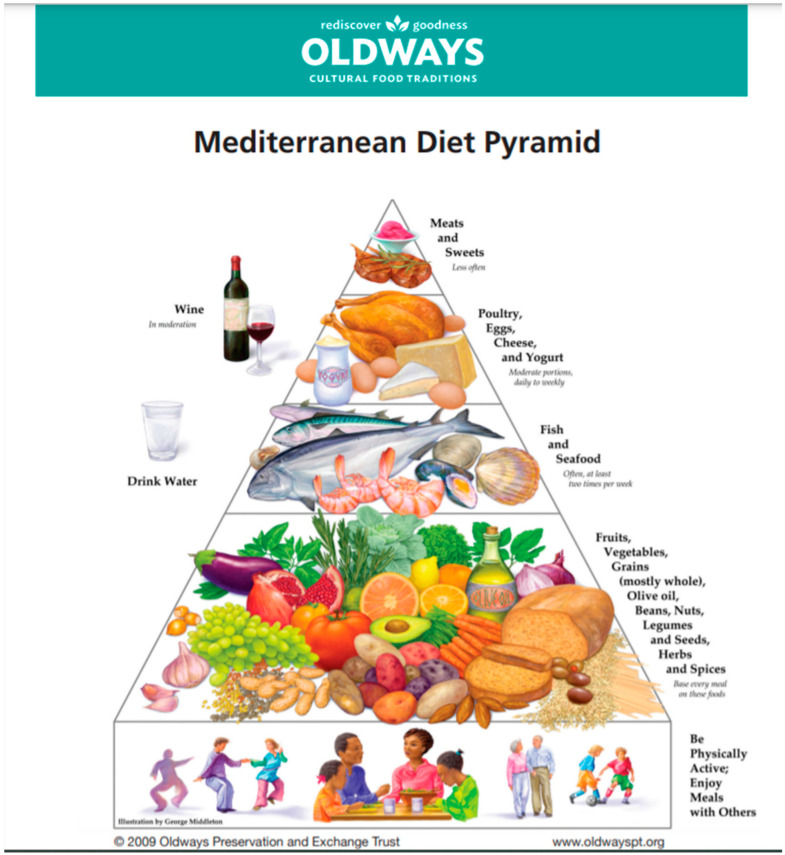
The initial concept for Mediterranean diet pyramid. In 1993, Oldways created the Mediterranean Diet Pyramid—in partnership with the Harvard School of Public Health and the World Health Organization (WHO).

The world “diet” can cause a lot of people to give up before they start. In popular culture, the word diet is associated with cutting entire food groups such as carbohydrates, fat, or dairy products. Alternatively, it can also mean making strict calorie restrictions. The popular Mediterranean diet is more than a strict meal plan, it should be adopted as a way of life [23]. It promotes seasonal cooking, freshly cooked meals, and use of extra-virgin olive oil. The Mediterranean lifestyle encourages people to socialize with each other, and to enjoy healthy and fresh food, in contrast to eating quick frozen meals prepared in the microwave, alone in front of the television. Residents of the Mediterranean region have followed the Mediterranean diet pattern and lifestyle for hundreds of years. The philosophy of the Mediterranean diet consists of eating more legumes, vegetables, fruits, nuts, wholegrain foods, and fish. To make it more approachable, the Mediterranean diet pyramid was introduced in 1993 [24]. This presentation appears to have endured, while giving guidance to millions of people globally. The food pyramid has undergone several changes [25], although it remains close to the original diagram (Figure 1). The popularity of the Mediterranean diet appears to have grown continuously, in both pop culture and the world of medicine.

### Mediterranean Diet in the Center of Clinical Studies

The term “Mediterranean diet” does not refer to a typical meal plan, on the contrary, it should be understood as a way of life. The original meaning of the Greek word “diatia” refers to a lifestyle [24,25]. Logically, the origins of the Mediterranean diet probably lie at the dawn of civilization, and these origins were typical of the people living in the Mediterranean region. However, at the end of World War II, Ancel Keys—who was a specialist in animal physiology and biology—introduced the world to the benefits of the Mediterranean diet and coined the term [26,27]. He contrasted the cardiovascular health of businessmen in the United States (who had a high incidence of heart attacks) to those populations where diets consisted of low fat and low calories, mostly as a side effect of the war. He also noted that there was a worsening of the trend of cardiovascular disease (CVD) after nations recovered from the war and started eating regular food, with more fat and more calories. Inspired by the opportunities that lay ahead, Keys traveled to Naples, Italy (where myocardial infarction was less common than in other developed countries), opened a portable laboratory, and discovered that Italians indeed had a low incidence of heart attacks and coronary disease [28,29]. During a convention of the World Health Organization in 1955 [30], his concept was questioned and challenged among international participants. Thus, the idea was born to implement a complex study [31] including seven countries, risk factors, and a follow-up of previously arranged parameters [32]; this approach is at the foundation of all studies in modern medicine. The results officially showed that the dietary habits implemented in traditional Mediterranean regions are associated with lower risk of cardiovascular events. However, there were and still are controversies over the clarity and originality of the data derived from this study. Many acknowledged scientists disapprove the “seven countries” study and oppose its findings. However, the popularity of the study is shown through the divided opinions among the scientific world even today.

However, despite the initial results and the success of the Keys study, the idea of the Mediterranean diet and Mediterranean lifestyle remained unrecognized until the end of the 20th century [33,34]. The next major study was the Lyon Diet Heart Study, which transformed some of the details of the French cuisine into typical Greek habits; for example, the more intensive use of olive oil [34,35,36,37]. The results showed a 50% reduction of new coronary episodes in patients who previously had acute myocardial infarction, in addition to a reduction in all causes of mortality [20,38,39]. This provided renewed interest in the possible health impact of the Mediterranean eating pattern, with a consequent permanent impact on medical research [34].

In subsequent years, the cardiovascular benefits of the Mediterranean diet became evident. The Spanish study originally named “Prevención con Dieta Mediterránea”, and globally known as PREDIMED, changed the course of related research [17]. Although the study was primarily outlined as a primary prevention randomized controlled trial, it proved that the Mediterranean lifestyle is closely associated with reductions in cardiovascular risk and disease; pro-atherogenic genes expression [37]; surrogate markers, such as waist-to-hip ratio; lipoprotein particles; lipid ratios; oxidative stress; and markers of inflammation [22,38]. Finally, it showed that the Mediterranean lifestyle reduces the risk of type 2 diabetes [20] and metabolic syndrome [40]. Despite its humble beginnings, PREDIMED revolutionized related medical research and became a widely recognized eating pattern among different populations, in addition to being acknowledged by medical experts regarding type 2 diabetes and its effects.

The Mediterranean diet was initially promoted as a dietary pattern with benefits for cardiovascular health. At that time, diabetes was regularly overlooked and understood as a predominantly hyperglycemic state. Due to these cardiovascular studies, however, it soon became clear that type 2 diabetes mellitus and cardiovascular health are closely intertwined. Thus, the Mediterranean diet was promoted by scientists as a potential solution to the global pandemic caused by type 2 diabetes.

## 4. Type 2 Diabetes and Mediterranean Diet

**Figure 2:**
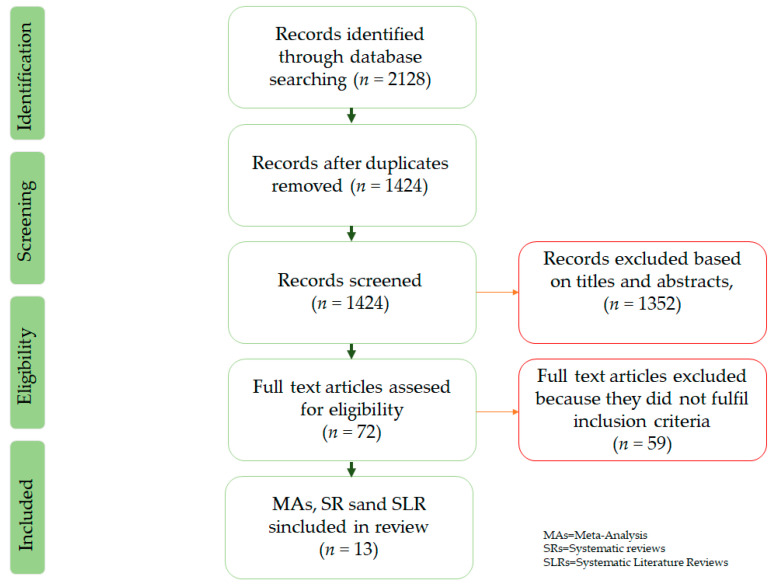
Flow diagram of literature search to identify Meta-Analysis and Systematic reviews evaluating the effect of Mediterranean diet on glycemic control in type 2 diabetes mellitus and diabetes prevention.

Due to the high incidence of type 2 diabetes, in addition to the increasing popularity of the Mediterranean lifestyle, our initial search revealed an extensive body of literature regarding these two topics. We conducted a structured internet literature search using PubMed and Google Scholar of articles published during the past seven years; specifically, we searched for medical articles published in the period from the publication of PREDIMED to June 2020. The search was concentrated on Meta-analyses, randomized controlled trials (RCTs), and systematic reviews (Figure 2). References of included publications were looked into, as well, for additional studies. The key search words and terms that we used were: type 2 diabetes, diabetic, Mediterranean diet, glycemic control, prediabetes, diabetes prevention, obesity, and insulin resistance. The publications we identified included hundreds of thousands of individuals that participated in prospective cohort studies and clinical trials.

### 4.1. Meta-Analysis, Structured Reviews and Randomized Controlled Trials

Table 1 presents all of the systematic reviews and meta-analysis from 2013 to 2020, which show the association between the Mediterranean diet and type 2 diabetes, and were used in this review.

A systematic review and meta-analysis of different dietary approaches to the management of type 2 diabetes conducted by Ajala et al. [41], published in 2013, indicates that different types of diet (Mediterranean, low glycemic index, low-carbohydrate, and high-protein diets) have a positive impact on people with diabetes, and that they are effectual in improving several markers of cardiovascular risk, which has high incidence in type 2 diabetes individuals. Their review evaluated the effects of several diets on HbA1c (referred to as glycemic control), weight loss difference, and changes in high-density lipoproteins (HDL) cholesterol, low-density lipoprotein (LDL) cholesterol, and triglycerides. The results indicated that all of the aforementioned diets led to an improvement in glycemic control. The Mediterranean diet, however, compared with other diets [52,53,54], showed a reduction in HbA1c of 0.47%, which indicated a significant improvement of glycemic control. In addition, the Mediterranean diet was also superior in achieving weight loss, with a weighted mean difference (WMD) in weight loss of 1.84 kg. Additionally, the Mediterranean diet significantly decreased triglycerides (WMD: −0.21 mmol/L) and improved HDL levels (WMD: +0.04 mmol/L). By reducing HbA1c, hence improving glycemic control, causing weight loss, and reducing triglycerides with the parallel rise of HDL, the Mediterranean diet presented itself as a superior diet regimen for patients with diabetes in this review [41].

A meta-analysis conducted by Carter P. et al. in 2013 presented results that indicated that the Mediterranean diet betters HbA1c, but not fasting blood glucose, when compared to alternative dietary patterns in those at risk of (or diagnosed with) diabetes [42]. This analysis, despite its limitations, underlined the need for further research in this area, because no firm conclusions were made.

Koloverou et al. carried out a meta-analysis in 2014 that studied the effect of the Mediterranean eating pattern on the development of type 2 diabetes mellitus. The findings indicated that greater adherence to the Mediterranean diet was associated with reduced risk of developing type 2 diabetes of up to 23%. This meta-analysis conveyed the need for an adjusted type of Mediterranean diet, depending on the local food availability and personal needs, to give individuals with a risk of developing type 2 diabetes broader and more variable nutritional choices for the purpose of primary prevention of the disease [43].

In 2014, Esposito K et al. published a meta-analysis of prospective cohort studies to assess the association between different diets and prevention of type 2 diabetes [44]. The purpose of the analysis was to evaluate the role of different diets in type 2 diabetes prevention, and to determine the risk of diabetes associated with healthy diets. The findings of the random-effect meta-analysis demonstrated that the risk of diabetes did not significantly change due to the geographical location, but the Mediterranean diet and dietary approach to stop hypertension (DASH) were closely associated with a 20% reduced risk of future type 2 diabetes. The Mediterranean diet was superior to the low-fat diet and the control group by reduction of HbA1c by 0.32% to 0.53%. However, the Mediterranean diet was not found to be superior compared to DASH [44]. Nonetheless, the results of the diet group intertwined comparison showed a 20% reduced risk of future type 2 diabetes, with a simple intermediation as a certain dietary pattern, including the Mediterranean diet.

Emadian A. et al. authored a systematic review of dietary randomized controlled trials in overweight and obese adults with type 2 diabetes in 2015 [45]. The results, however, did not show a significant difference in weight loss between treatment groups. Despite the fact that there was no general consensus, this systematic review showed studies that favored the Mediterranean diet compared to other diets, such as the ADA conventional and low-fat diets [45,55,56].

Another systematic review by Esposito K. in 2015 compared the effect of the Mediterranean diet with a control diet (mainly low-fat diet) on the treatment of type 2 diabetes and prediabetes, with the outcomes being glycemic control, cardiovascular risk factors, and metabolic syndrome remission. The gathered data conclusively showed that the Mediterranean diet was associated with better glycemic control and cardiovascular risk factors than the control diets, and that the Mediterranean diet is suitable for the overall management of type 2 diabetes [46].

Huo R. et al. published a meta-analysis concentrating on the effect of the Mediterranean diet on glycemic control, cardiovascular risk factors and weight loss among type 2 diabetes individuals [47]. The results clearly demonstrated the clear advantage provided by the Mediterranean diet comparted to other types of diet. When compared to other diets, the Mediterranean-style diet showed greater reductions in HbA1c with a mean difference (MD) of −0.30%, greater reductions of fasting plasma glucose with MD of −0.72 mmol/L, greater reductions of fasting insulin with MD of −0.55 μU/mL, significant reductions of body mass index with MD of −0.29 kg/m^2^, and reductions of body weight with MD of −0.29 kg. Similarly, concentrations of total cholesterol and triglyceride were reduced (−0.14 mmol/L and −0.29 mmol/L, respectively), and high-density lipoprotein increased with MD of +0.06 mmol/L. In addition, the Mediterranean diet was associated with a decline of 1.45 mm Hg for systolic blood pressure and 1.41 mm Hg for diastolic blood pressure. The results indicated that the Mediterranean diet not only improved glycemic control and weight loss, but it also played a significant role in ameliorating the lipid profile and blood pressure in people with type 2 diabetes mellitus [47]. This meta-analysis clearly demonstrated to the medical profession that the Mediterranean diet should have a more prominent role in the management of type 2 diabetes.

Schwingshackl L. et al. conducted a meta-analysis on more than 100,000 subjects, regarding the adherence to a Mediterranean diet and its possible health improvements. The analysis revealed a significant interdependence between adherence to the dietary pattern and an overall decrease in the risk of type 2 diabetes mellitus of 19% [19].

In 2017, Jannasch F. et al. inspected the connection between certain dietary habits and type 2 diabetes [16]. This systematic review and meta-analysis showed that the Mediterranean diet, alternate healthy eating index (AHEI), and DASH each show potential to help prevent diabetes, with differences in the particular components. The effect of the Mediterranean diet on diabetes prevention varied from 9% to 20%, depending on the study, and depending on the type of Mediterranean diet that was involved. The results suggested that the greater the Mediterranean diet score, the greater the grade of risk reduction (risk ratio for comparing extreme quantiles: 0.87; 95% CI: 0.82, 0.93) [16].

A thorough systematic meta-review by Martinez-Lacoba R. et al. summarized and synthesized the effects of the Mediterranean diet on different health outcomes. The meta-review included (among other parameters): adherence to the Mediterranean diet, cardiovascular disease (CVD), health related quality of life, hypertension, metabolic syndrome, obesity, body weight and body mass index (BMI), and type 2 diabetes. Among other findings, the synthetized results showed that the Mediterranean diet has a beneficial impact on body weight, type 2 diabetes prevention, and metabolic syndrome [48].

Schwingshackl L. conducted another meta-analysis in 2018 that focused on the comparison of the efficacy of different dietary patterns on glycemic control in individuals with type 2 diabetes [49]. The analysis included nine dietary approaches—Mediterranean, low-fat, vegetarian, high-protein, moderate-carbohydrate, low-carbohydrate, control, low glycemic index (Low GI), and Paleolithic. “The results were in favor of all of the dietary patterns, meaning that all of them improved the glycemic control, by significant reduction of HbA1c (−0.82 to −0.47%) and significant reduction of fasting glucose (−1.61 to −1.00 mmol/L) compared to a control diet. The Mediterranean (MD: −0.32, 95% −0.53, −0.11) and the LC diet (MD: −0.35, 95% −0.56, −0.14) were more effective in reducing HbA1c compared to a LF diet. The Mediterranean diet was more effective in reducing fasting glucose compared to a LF (MD: −0.61 mmol/L, 95% −1.03, −0.20) and LGI/GL diet (MD: −0.59 mmol/L, 95% −1.13, −0.04).” In summary, the Mediterranean diet was shown to be superior overall compared to the other dietary approaches by reducing the HbA1c (80%) and the fasting plasma glucose (88%), i.e., the surface under the cumulative ranking curve (SUCRA) value [49].

Mingyue Zeng et al. authored a review of six RCTs, which concentrated on the Mediterranean diet and its effects on the prevention of type 2 diabetes amongst overweight patients. Results showed that adopting a low-carbohydrate Mediterranean diet or a Mediterranean diet with virgin olive oil (with regular physical activity) has a positive impact on the possible prevention of type 2 diabetes in overweight patients. However, due to limitations outlined by the author, there is an urgent need for more prolonged and detailed studies regarding obese adults and type 2 diabetes prevention [50].

A detailed meta-analysis published by Becerra-Tomas N. in 2019 suggested that the Mediterranean diet has an important role in cardiovascular disease prevention in patients with type 2 diabetes. The results showed that the Mediterranean diet has a beneficial effect on total CVD incidence in patients with diabetes, and total myocardial infarction incidence in people with type 2 diabetes. Furthermore, it showed that the group with the highest adherence to the Mediterranean diet showed the lowest association with overall CVD mortality [51].

### 4.2. Mediterranean Diet as a Nutritional Therapy for Type 2 Diabetes

Regardless of the type of study, year of publication, or number of participants, all of the abovementioned analyses and reviews provide general support for the Mediterranean diet as an important tool in the prevention and treatment of type 2 diabetes. The recommendations for nutritional therapy made by the ADA are focused on maintaining a healthy weight, while sustaining optimum levels of HbA1c, blood pressure, and lipid fractions [7,14]. To achieve these goals, people at risk of, or those with, diabetes need a dietary pattern that would serve them as a nutritional therapy, while also enabling them to gain the benefits of the diet. It would appear that the Mediterranean lifestyle is included in this category [57].

Strict glycemic control is valuable in terms of preventing diabetes complications in the long term, as shown previously in the United Kingdom Prospective Diabetes Study (UKPDS) [58]. The Mediterranean diet has a beneficial effect on HbA1c, and this reduction varies between analyses, depending on the period of intervention, the number of participants, and the diet used as a comparison group. However, the Mediterranean diet has consistently been shown to have a beneficial impact on HbA1c by reducing it from between 0.3% and 0.47% [41,45,46,47,49]. In addition, data gathered in meta-analyses shows that constancy to the Mediterranean eating pattern results in a decrease in glycemic levels, with a MD of −0.59 mmol/L [49].

Diabetes treatment is broader than only glycemic control, and encapsulates healthy weight and a healthy lipid profile, which could lead to reduced risk of cardiovascular and coronary heart disease, including myocardial infarction, stroke, and atherosclerosis. Diabetic dyslipidemia consists of elevated triglycerides and LDL cholesterol, low HDL-cholesterol, and a preponderance of small dense LDL particles [59]. This lipid pattern represents the paramount link between diabetes and the inflated cardiovascular risk of diabetic patients, making it the leading cause of death in individuals with diabetes [60]. Thus, the benefits of a certain dietary pattern for individuals with type 2 diabetes must include more than a lowering of the percentage of HbA1c. The data gathered in the reviewed studies indicates that the Mediterranean diet meets these requirements regarding a nutritional therapy for patients with diabetes. The Mediterranean diet is also effective in ameliorating the lipid profile. By decreasing triglyceride levels (−0.29 and −0.21 mmol/L) and increasing HDL (+0.04 and +0.06 mmol/L), the Mediterranean diet is superior in diabetic patients to other dietary approaches [41,47]. The benefits of the Mediterranean diet are also shown by the reduction in the incidence of total cardiovascular disease (CVD) and myocardial infarction in patients with diabetes [51]. The same meta-analysis presented an inverse association of the highest versus lowest categories of Mediterranean diet adherence, with total CVD mortality, stroke, and coronary heart disease incidence. Adherence to the Mediterranean diet also results in weight loss, and sustaining a healthy weight plays a significant role in diabetes treatment and prevention. Furthermore, the evidence gathered from different studies shows that the Mediterranean diet results in significant weight loss, which depends on the level of adherence and the period of intervention. Groups that had an intervention of about 6 months or longer lost weight, with a MD of −1.84 [41], whereas groups that had intervention period of two years or longer lost from 4.1 to 10.1 kg [61].

In addition, the collected data indicates that adherence to the Mediterranean diet also has a favorable effect on diabetes prevention. Several publications suggested that the greater the adherence, the larger the reduction of diabetes, with reductions varying from 23% to 59% [16,19,21,42,56].

### 4.3. Nutritional Aspect of the Mediterranean Diet and Its Effect on Type 2 Diabetes

**Figure 3:**
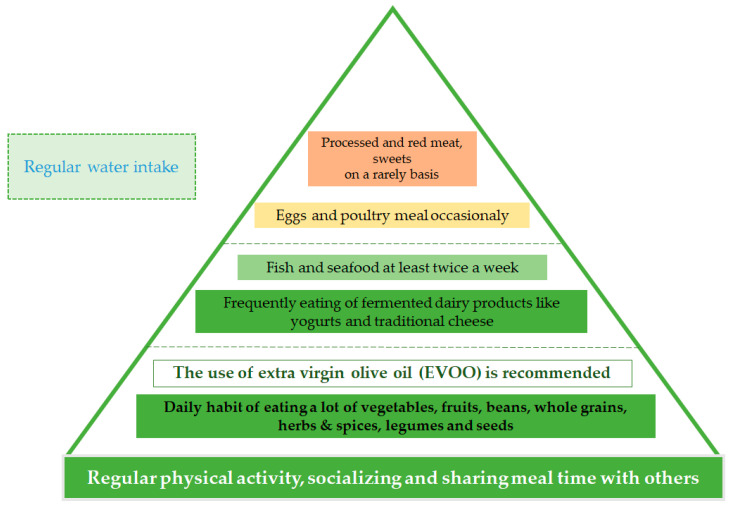
The foundation of the Mediterranean diet based on the original recommendation created by Oldways in partnership with the Harvard School of Public Health and the World Health Organization.

Despite the ongoing changes in the pyramid of the Mediterranean diet, certain baseline characteristics of this diet have not changed (Figure 3). The United Nations Educational, Scientific and Cultural Organization (UNESCO) recognized the Mediterranean diet by putting it on the “Representative List of the Intangible Cultural Heritage of Humanity” in 2013, and therefore establishing it as a dietary pattern with notable characteristics [62].

The base of this pyramid consists of a variety of unprocessed whole grains, legumes, cereals, fresh fruits, and vegetables, and dried fruits, nuts, and seeds are identified as daily healthy snacks. The main source of fat is extra-virgin olive oil (EVOO), which is low in saturated fat, and rich in poly and mono saturated acids. Sea food and poultry, and unprocessed cheese and yogurt, belong to the group of low to moderate consumption. Red and processed meats, and sweets, are at the top of the pyramid, with a recommendation for very low consumption.

The Mediterranean diet positively affects microbiome diversity, and lowers oxidative stress, LDL levels, and inflammation, while improving insulin sensitivity and immune function. Consequently, it reduces type 2 diabetes mellitus, obesity, metabolic syndrome, and CVD [63]. Research at the cellular level of the Mediterranean diet indicates that it is rich in fibers, mono-saturated fatty acids (MUFA) and poly-saturated acids (PUFA), probiotics, low glycemic foods, vitamins, and antioxidants. This specific content positively affects lipid profile, obesity, and the associated inflammatory state [22,64,65]. The Mediterranean nutritional profile is efficient for prevention and glycemic control of type 2 diabetes [49,51,66,67]. Hyperglycemia, which can be common in type 2 diabetes, leads to increased intracellular oxidative stress and subsequent overproduction of free radicals. The endogenous antioxidants present in the Mediterranean diet lead to a significant decline in the content of free radicals, and therefore prevent or reduce the damaging effects of chronic hyperglycemia [68]. The MUFA improve the postprandial lipid and GLP-1 responses in insulin resistant subjects [69]. The Mediterranean diet also improves gut immune function, and reduces gut leakiness and endotoxemia [63,64].

The Mediterranean diet pyramid can be assessed at each level to provide clear data that intertwines the diet with improvements in glycemic control in patients with diabetes or reductions in the risk of developing type 2 diabetes [70]. Regular uptake of whole grains plays a significant role in the prevention of type 2 diabetes mellitus [71] by lowering post-prandial blood glucose levels and insulin resistance in obese adults [72], and by lowering post-prandial blood glucose and insulin and the maximal glucose and insulin response in healthy subjects [70,73]. Frequent use of vegetables in the dietary pattern, although minimal, nonetheless reduces the risk of type 2 diabetes development [70,71]. Daily intake of different fruits as a part of the Mediterranean diet is beneficial to type 2 diabetes mellitus [70,71,74]. Vegetables and fruits are rich in fibers and antioxidants that improve type 2 diabetes prevention by reducing the risk of weight gain and improving insulin sensitivity [70,74,75,76]. Nuts and seeds, which are recommended as daily snacks in the Mediterranean diet, reduce oxidative stress and improve the endothelial function [70,77], therefore improving the lipid profile and reducing insulin resistance [78]. Small daily portions of yogurt have been shown to have a significant reduction capability regarding the risk of type 2 diabetes mellitus [42,70,79,80,81]. More specifically, low fat dairy products play a greater role in the prevention of type 2 diabetes mellitus [42]. Red and processed meats, and desserts with high carbohydrate content, which are at the top of the pyramid with the lowest intake recommendation, are associated with an increased risk of type 2 diabetes mellitus [70,71].

Olive oil, which represents the authentication of the Mediterranean diet, has a significantly positive effect on type 2 diabetes mellitus [70]. Thus, the use of olive oil and the possible effects it may have on the metabolism, and consequently on the prevention or treatment of type 2 diabetes mellitus, is central to the discussion of the benefits of the Mediterranean diet. Olive oil decreases the risk of type 2 diabetes mellitus by 13% [71], ameliorates the lipid profile, and has beneficial roles regarding low degree inflammation and endothelial function [82,83,84]. A one-year intervention study by Hernáez et al. showed that when the Mediterranean diet is enhanced with virgin olive oil, it improves several “HDL functions such as cholesterol efflux capacity, cholesterol metabolism, anti-oxidant/anti-inflammatory properties, and vasodilatory capacity in individuals at high cardiovascular risk” [85,86]. Data from another study, by Covas, indicates that after consuming phenolic olive oils, the levels of HDL-C increase, with a significant drop in the levels of total cholesterol, triglycerides, and LDL, and a concomitant decrease in total cholesterol/HDL and LDL/HDL ratio [85,87]. Moreover, the use of olive oil appears to improve endothelial function in patients with prediabetes and diabetes, as shown in a report about Coronary Diet Intervention with Olive Oil and Cardiovascular Prevention, or the “CORDIOPREV” study [88,89]. The results from this report presented evidence that after an intervention of 1.5 years with a Mediterranean diet rich in EVOO, there was an improvement in flow-mediated vasodilatation in type 2 diabetes individuals (5.2 ± 0.4 at 1.5 years vs. 3.8 ± 0.4 at baseline; *p* = 0.04) [88,89]. Given the high cardiovascular risk of type 2 diabetes patients, this study shows that the Mediterranean diet rich in EVOO could represent a strong basis for future treatment of type 2 diabetes.

In addition to improving the endothelial function and the lipid profile, a Mediterranean diet enriched with EVOO decreases the postprandial blood glucose and increases the insulin levels, and therefore has a favorable impact on the glycemic outline in healthy subjects [77]. Olive oil is naturally rich in polyphenols, and studies suggest that these polyphenols might uniquely affect the glucose metabolism by inhibiting the digestion and therefore absorption of carbohydrates, followed by reduced levels of blood sugar delivery from the liver, or stimulation of blood sugar uptake in peripheral tissues [85]. Due to their antioxidative properties, the polyphenols might deplete the production of advanced glycosylated end products and lead to reductions in the glycemic load by gradually decreasing the hyperinsulinemia to normal levels with a parallel improvement in insulin sensitivity [90].

### 4.4. Mediterranean Diet vs. Insulin Resistance

**Figure 4:**
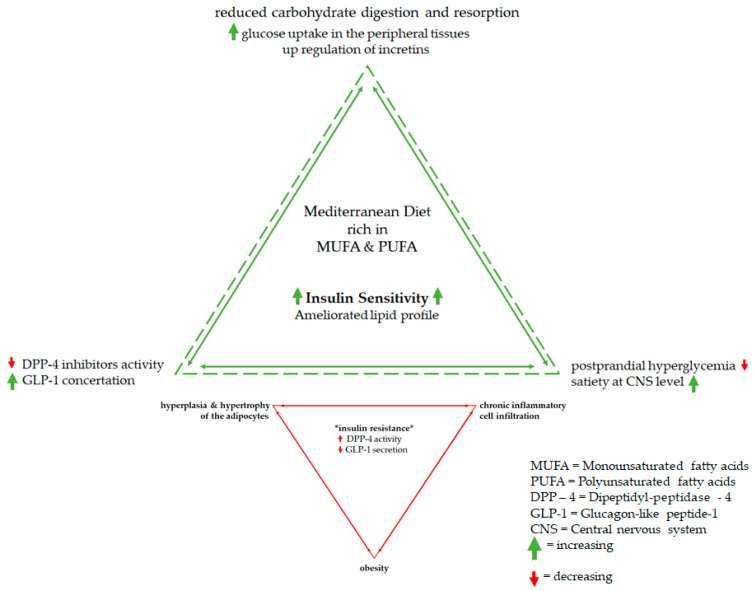
Mediterranean diet vs. Insulin resistance, possible mechanisms.

Insulin resistance has become the most common metabolic state affecting millions of individuals of various population and age groups, including obese individuals and women with polycystic ovarian syndrome. The most recurrent outcome of insulin resistance globally is type 2 diabetes [12]. Obese individuals are affected by hyperplasia and hypertrophy of the adipocytes (cells in adipose tissue), thus making them dysfunctional, and causing chronic inflammatory cell infiltration, which leads to activation of the complex cytokines network. This constitutes the main factor in developing insulin resistance and consequently developing type 2 diabetes [91,92]. Individuals that have insulin resistance are at extremely high risk of developing type 2 diabetes. Nonetheless, this risk can be mitigated by interventions based on simple lifestyle changes. This includes the Mediterranean eating pattern, which is linked with positive outcomes in clinical research conditions regarding insulin resistance [20], the potential mechanism is shown in Figure 4.

Monounsaturated fatty acids (MUFA) and polyunsaturated fatty acids (PUFA), in particular, are highly present in the Mediterranean diet and can be found in diverse forms in olive oil, nuts, and seeds. Furthermore, they are central to the refinement of the glucose metabolism, the improvement of insulin sensitivity and lipid profile, and the parallel reduction of CVD risk. As shown in reports from PREDIMED, the overall risk of type 2 diabetes can be reduced even in cases in which calories are not restricted [20]. The protective and apparently antidiabetic effects of MUFA and PUFA were neatly presented in a meta-analysis by Quain F. et al. [93]. The data from this meta-analysis showed enhanced glycemic control, improved lipid levels, and decreased systolic blood pressure levels in individuals with diabetes. In a prospective cohort study by Martínez-González et al., published in 2008, evidence suggests that the increased intake of MUFA or PUFA on a daily basis, mainly through olive oil and seeds rather than saturated or trans fatty acids, may significantly lower the risk of type 2 diabetes by up to 83% over a mean time period of 4.4 years [67,94]. It is considered that PUFA may refine the inflammatory responses of the dysfunctional adipose tissue, hence resulting in favorable effects on insulin sensitivity [95]. The probable mechanisms are reduced carbohydrate digestion and resorption, and/or greater glucose uptake in the peripheral tissues, which takes place in a complex process involving up-regulation of incretins, which is then followed by reduced dipeptidyl peptidase-4 activity, and a consequential rise in the concentration of GLP-1 [96]. The aforementioned elevated levels of GLP-1 may limit postprandial hyperglycemia and influence satiety at the central nervous system level [95]. As a result of the diminished glycemic load, there is a reduced demand for insulin, which leads to greater insulin sensitivity [93,97].

## 5. Discussion

Diabetes is regarded as a major health issue, and has unfortunately reached concerning levels, rapidly exceeding statistical expectations. Today, near half a billion people have diabetes globally, with 90% of them being type 2 diabetes mellitus cases. An apparent pathognomonic characteristic of diabetes is that two-thirds of people suffering from the disease live in urban areas and three-quarters are of working age [12]. These statistics highlight the negative effects of diabetes on overall health globally, indicating significant potential for short- and long-term complications, and implications for the daily lives of affected individuals. A systematic global improvement in diabetes is required, including a tool that is accessible to all those affected, or at risk, to suppress the diabetes epidemic.

The Mediterranean diet has continuously proven to be a valuable contributor to the practical treatment of type 2 diabetes mellitus. Official publications have repeatedly shown that adherence to the Mediterranean diet results in HbA1c reduction, reduced risk of cardiovascular and coronary heart disease, improvement of diabetic dyslipidemia, and weight loss. Furthermore, information gathered in the past indicates that adherence to the Mediterranean diet also has a favorable effect on diabetes prevention. Due to all of these components, the Mediterranean diet largely fulfills the recommendations for nutritional therapy of the ADA [7,14]. Most of the associated controversy, if any, relates to the fact that the Mediterranean diet has consistently been found to be superior to control groups, but has failed to show superiority compared to DASH, AHEI, and Paleolithic diets (or, in some cases, vegetarian diets) [16,42,44,45]. This, however, does not invalidate the beneficial effects of the Mediterranean diet. Rather, it indicates the need for bigger and longer clinical trials, conducted on different continents, which will prove the appropriateness and flexibility of the Mediterranean diet based on local opportunities.

Recent years have shown a sharp increase in glucagon like peptide 1 (GLP-1) receptor agonists [98], sodium-glucose co-transporter-2 (SGLT2) inhibitors [99], thiazolidinediones [100], and dipeptidyl peptidase 4 (DPP-4) inhibitors [101]. Each of these medications revolutionized diabetes treatment by providing new mechanisms to counter the disease using various approaches and practically mimicking healthy non-diabetic organisms. The synergetic effects of the Mediterranean diet at the cellular level raise the question of whether all the pharmaceutical studies have been based on the possible pathways that lie hidden in the Mediterranean diet.

The diabetes prevalence is on constant high level on the Balkan Peninsula, whether it may be because of bad eating habits, or lack of physical activity, the number of individuals with diabetes is rising. More precisely, in 2014 the national diabetes prevalence in Republic of North Macedonia was 11.44% if the adult population [102], and there is a growing prevalence and incidence of diabetes in the past five years [103]. According to the data presented by the IDF [12] the prevalence of diabetes in the rest of the Balkan countries is moderate to high: there is a 12% prevalence of diabetes in adults in Serbia, 11.7% in Bosnia and Herzegovina, 6,8% diabetes prevalence in Croatia, 11.1% in Albania, 8.3% in Bulgaria, 7.4% in Greece, and 7.8% in Slovenia [12]. Certainly, this is a result of the significantly increased caloric intake over the past two decades, and it is important to mention that in this time period most of the Balkan countries were or still are in social transition and belong to low or middle income countries. The transitioning period is still ongoing in North Macedonia, Serbia, and Bosnia and Herzegovina, and as mentioned before these countries have high diabetes prevalence. The increased caloric intake is mostly in form of easily available and cheap meals, such as types of flour based breads, cheap animal products (mostly pork, beef, and fatty cheese) and overuse of sunflower oil [102]. With this in mind, there is an urgent need on the Balkans for healthy eating pattern that will be available and affordable for the majority of the population, in order to sustain the rapid rising of diabetes cases. Despite all of the typical bad eating patterns adopted by the Balkan people, the climate on the Balkan Peninsula is very tame and a lot of vegetables and fruits grow here. The possibility for homegrown vegetables and fruits makes them affordable for the local population, and it lowers the need for import of different types of fast food as part of the Westernized dietary habits, which could also be beneficial for the environment as well.

The environmental impact represents a major part of every dietary pattern in different regions of the world. There is not enough data yet, but the available scientific literature suggests that implementing certain dietary patterns can cause lesser environmental impact than the current environmental footprint left by the mass consumption societies [104] Specifically, adherence to the Mediterranean eating pattern could be beneficial, considering the idea that the Mediterranean diet pyramid is based of everyday intake of fruits, vegetables and legumes, with low consumption of processed meats. With locally produced vegetables and fruits, based on the actual need of a certain country population, the environmental impact could improve. Most of the known dietary patterns (Mediterranean diet, DASH, AHEI, Low carb, and Low fat) are a preferable option to the “Westernized” diets based on mass produced, cheap and easily available high caloric meals, with an end result of piles of waste. Locally grown products and sustaining everyday needs with local production and lesser use of processed meats, as a basis for a certain dietary pattern could have important part in lowering the carbon footprints.

## 6. Conclusions

The uniqueness of the Mediterranean diet lies in the ease with which its main components sustain diabetes homeostasis, whether by improving insulin sensitivity and the gut microbiome, or by stimulating anti-inflammatory and antioxidant actions. The simple ingredients of this centuries-old dietary pattern apparently continue to baffle scientists, and could possibly be revolutionary for treatment of the “plague of the 21st century”.

## Figures and Tables

**Table 1:** Systematic reviews and meta-analysis of the association between the Mediterranean diet and type 2 diabetes (T2D) 2013–2020.

Author, Year of Publication, Type of Study, Reference	Intervention, Participants	Evaluation, Follow Up	Results/Conclusion
Ajala, O. 2013, 20 RCTs [41]	Low carbohydrate, Low GI, MedDiet, High protein diet Adults with type 2 diabetes or obesity	HbA1c Weight loss, >6 months	All diets improved glycemic parameters (glycated hemoglobin reductions of −0.12% LC (*p* = 0.04), −0.14% Low GI (*p* = 0.008), −0.47% MedDiet (*p* < 0.00001), and −0.28% High protein (*p* < 0.00001)). Low-carbohydrate and MedDiet led to significant loss in weight (−0.69 kg (*p* = 0.21) and −1.84 kg (*p* < 0.00001), respectively). Increase in HDL was noted in all diets except the high-protein diet.
Carter, P. 2014, SR, MA 8 RCTs, [42].	MedDiet, Paleo diet, Control diet Overweight and/or high cardiovascular risk and/or type 2 diabetes	Glycemic control, HbA1c, Insulin, 2–12 months	MedDiet superior compared to the control group, except when compared to the paleo diet. None of the diets proved preferable regarding the basal glucose levels.
Koloverou, E. 2014, SR, MA 1 RCT, 9 prospective studies [43].	MedDiet, Control diet Healthy adults with or without CV/T2D	Incidence of type 2 diabetes, 3.5–14 years	Higher adherence to the MedDiet was associated with 23% reduced risk of developing type 2 diabetes (combined relative risk for upper versus lowest available centile: 0.77; 95% CI: 0.66, 0.89)
Esposito, K. 2014, MA 8 prospective studies, 30 cohorts. [44].	MedDiet, Dietary Approach to Stop Hypertension (DASH) Adults	Incidence of type 2 diabetes, 3.2–23 years	Both diets presented themselves as a healthy dietary pattern, however comparison between MedDiet and. DASH showed no changes in incidence of type 2 diabetes.
Emadian, A. 2015, SR 11 RCTs [45].	MedDiet, Vegan diet, Low glycemic index diet Overweight adults and type 2 diabetes	Glycemic control, HbA1c, >6 months	All diet groups resulted beneficial, with improved overall glycemic control by reduction of HbA1c levels.
Esposito, K. 2015, SR 8 MA, 5 RCTs [46]	MedDiet Control diet Adults with type 2 diabetes or at risk	Incidence of type 2 diabetes Glycemic control, >6 months	MedDiet improves glycemic control by reducing HbA1c 0.3–0.47% compared with low-fat diet. MedDiet has a significant reduction of incidence of future type 2 diabetes ranging from 19% up to 23%
Huo, R. 2015, MA 9 RCTs [47].	MedDiet Adults with type 2 diabetes	Glycemic control. HbA1c, insulin, Homeostatic Model Assessment for Insulin Resistance (HOMA), 1 month–4 years	The MedDiet group had a significant reduction of HbA1c levels (MD−0.30; 95% CI −0.46, −0.14), glucose levels (MD −0.72 mmol/L; CI −1.24, −0.21), and baseline insulin levels (MD −0.55 μU/mL; CI −0.81, −0.29).
Schwingshackl, L. 2015, SR, MA 1 RCT, 8 prospective studies [19].	MedDiet Healthy adults or with CV risk factors	Incidence of type 2 diabetes, 3.2–20 years	Adherence to MedDiet inversely associated with a decrease in T2D incidence (high vs. low. RR: 0.81; 95% CI 0.73, 0.90, *p* < 0.0001)
Jannasch, F. 2017, SLR, MA 48 articles compromising 16 cohorts/ACRs [16].	MedDiet, DASH, Alternate Healthy Eating Index (AHEI) Healthy adults	Incidence of type 2 diabetes, 4.1–23 years	Adherence to MedDiet (RR quantiles: 0.87; 95% CI: 0.82, 0.93), DASH (RR: 0.81; 95% CI: 0.72, 0.92), and AHEI (RR: 0.79; 95% CI: 0.69, 0.90) associated with a significant risk reduction of type 2 diabetes
Martinez-Lacoba, R. 2018, SMR 9 SR, 24 MA [48]	MedDiet Adults	Diet adherence, obesity, body weight type 2 diabetes, >6 months	In summary, a MedDiet pattern may help to prevent and manage type 2 diabetes.
Schwingshackl, K. 2018, SR, MA 56 RCTs [49]	Low-fat diet or vegan, MedDiet, LC, paleolithic hyperprotein diet Adults with T2D	Glycemic control HbA1c Weight change, 3–48 months	The MedDiet overall superior when compared to the other dietary approaches, by reducing the HbA1c (80%) and reducing the fasting plasma glucose (88%).
Zheng M. 2018 SR, 6 RCTs [50]	Modified MedDiet Adults with obesity	Glycemic control HbA1c, >3 months	Low-CHO MedDiet and MedDiet using virgin olive oil had a positive impact on the prevention of overweight patients within T2D
Becerra-Tomas, N. 2019, SR, MA 3 RCTs, 38 cohorts, [51].	MedDiet Adults with type 2 diabetes	CVD incidence, myocardial infarction, CVD mortality, coronary heart disease incidence, >6 months	Beneficial effect on total CVD incidence (RR: 0.62; 95% CI: 0.50, 0.78) and total myocardial infarction (MI) incidence (RR: 0.65; 95% CI: 0.49, 0.88). Highest versus lowest categories of MedDiet adherence, revealed an inverse association with total CVD mortality (RR: 0.79; 95% CI: 0.77, 0.82), coronary heart disease (CHD) incidence (RR: 0.73; 95% CI: 0.62, 0.86), CHD mortality (RR: 0.83; 95% CI: 0.75, 0.92), stroke incidence (RR: 0.80; 95% CI: 0.71, 0.90), stroke mortality (RR: 0.87; 95% CI: 0.80, 0.96) and MI incidence (RR: 0.73; 95% CI: 0.61, 0.88).

MedDiet = Mediterranean diet, GI = glycemic index, LC = low carb, DASH = dietary approach to stop hypertension, AHEI = alternate healthy eating index, CHO = carbohydrate CVD = cardiovascular disease, RR = risk ratio, CHD = coronary heart disease, MI = Myocardial infarction, HbA1c = glycated hemoglobin A1c, MD = mean difference, MA = meta-analysis, SR = structured review, RCT = randomized control trial; SMR = systematic meta-review.

## Data Availability

No new data were created or analyzed in this study. Data sharing is not applicable to this article.
